# Longitudinal alterations in gamma-aminobutyric acid (GABA_A_) receptor availability over ∼ 1 year following traumatic brain injury

**DOI:** 10.1093/braincomms/fcac159

**Published:** 2022-06-15

**Authors:** Y Kang, K Jamison, A Jaywant, K Dams-O’Connor, N Kim, N A Karakatsanis, T Butler, N D Schiff, A Kuceyeski, S A Shah

**Affiliations:** Department of Mathematics, Howard University, Washington, DC 20059, USA; Department of Radiology, Weill Cornell Medicine, 407 E. 61 St., Rm 208, New York, NY 10065, USA; Department of Rehabilitation Medicine, Weill Cornell Medicine, New York, NY 10065, USA; Department of Psychiatry, Weill Cornell Medicine, New York, NY 10065, USA; Department of Rehabilitation and Human Performance, Icahn School of Medicine at Mount Sinai, New York, NY 10029, USA; Department of Neurology, Icahn School of Medicine at Mount Sinai, New York, NY 10029, USA; Department of Radiology, Weill Cornell Medicine, 407 E. 61 St., Rm 208, New York, NY 10065, USA; Department of Radiology, Weill Cornell Medicine, 407 E. 61 St., Rm 208, New York, NY 10065, USA; Department of Radiology, Weill Cornell Medicine, 407 E. 61 St., Rm 208, New York, NY 10065, USA; Department of BMRI & Neurology, Weill Cornell Medicine, New York, NY 10065, USA; Department of Radiology, Weill Cornell Medicine, 407 E. 61 St., Rm 208, New York, NY 10065, USA; Department of Radiology, Weill Cornell Medicine, 407 E. 61 St., Rm 208, New York, NY 10065, USA; Department of BMRI & Neurology, Weill Cornell Medicine, New York, NY 10065, USA

**Keywords:** flumazenil PET, cognition, traumatic brain injury, anterior forebrain mesocircuit, GABA_A_

## Abstract

Longitudinal alterations of gamma-aminobutyric acid (GABA_A_) receptor availability following traumatic brain injury have remained uncharacterized and may reflect changes in neuronal structure and function linked to cognitive recovery. We measured GABA_A_ receptor availability using the tracer [11C]flumazenil in nine adults with traumatic brain injury (3–6 months after injury, subacute scan) and in 20 non-brain-injured individuals. A subset of subjects with traumatic brain injury (n = 7) were scanned at a second chronic time-point, 7–13 months after their first scan; controls (n = 9) were scanned for a second time, 5–11 months after the first scan. After accounting for atrophy in subjects with traumatic brain injury, we find broad decreases in GABA_A_ receptor availability predominantly within the frontal lobes, striatum, and posterior-medial thalami; focal reductions were most pronounced in the right insula and anterior cingulate cortex (p < 0.05). Greater relative increase, compared to controls, in global GABA_A_ receptor availability appeared between subacute and chronic scans. At chronic scan (>1 year post-injury), we find increased pallidal receptor availability compared to controls. Conversely, receptor availability remained depressed across the frontal cortices. Longitudinal improvement in executive attention correlated with increases in receptor availability across bilateral fronto-parietal cortical regions and the anterior-lateral aspects of the thalami. The specific observations of persistent bi-frontal lobe reductions and bilateral pallidal elevation are consistent with the anterior forebrain mesocircuit hypothesis for recovery of consciousness following a wide range of brain injuries; our results provide novel correlative data in support of specific cellular mechanisms underlying persistent cognitive deficits. Collectively, these measurements support the use of [11C]flumazenil to track recovery of large-scale network function following brain injuries and measure response to therapeutics.

## Teaser

Kang *et al*. report that following traumatic brain injury, decreased GABA_A_ receptor availability is seen in the anterior forebrain mesocircuit. Recovery during the ∼1 year post is marked by widespread increases, especially in the pallidum, but remains depressed across the frontal cortices possibly contributing to enduring cognitive impairments.

## Graphical Abstract

**Graphical abstract:**
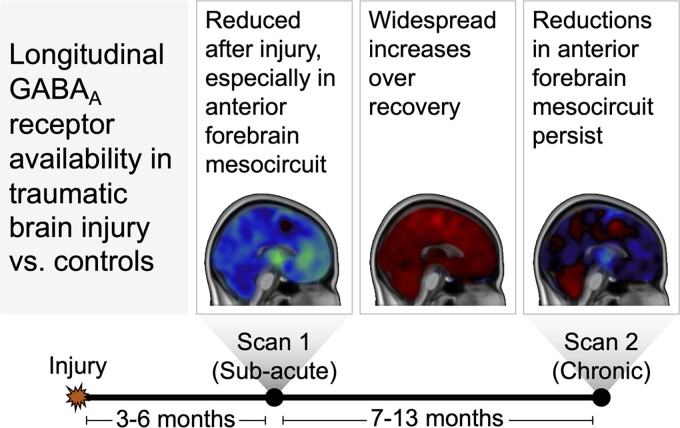

## Introduction

Traumatic brain injury (TBI) is a leading cause of death and long-term disability in the United States, as well as world-wide,^1^ and there are more than 5.3 million persons in the US alone living with chronic cognitive dysfunction.^2^ Impaired attention is the most common and debilitating cognitive deficit, contributing to long-term disability and barriers for community reintegration, functional independence, and productive employment.^2–4^ Chronic deficiencies in executive attention, such as the capacity to block distractors and selectively focus on goal-relevant tasks,^5^ have been noted.^6,7^ Neuroimaging studies have linked executive attention impairments to focal cortical and white matter damage in the medial frontal,^8,9^ fronto-parietal,^10^ fronto-striatal,^11^ and thalamic regions^12–14^ following TBI. However, these correlations with structural damage^8,10,11^ and electrophysiological responses,^9^ alone, do not provide insight into the underlying neuronal substrate associated with executive attention deficits. Most importantly, these measurements are incapable of directly measuring the alterations and dynamics of neuronal integrity within cortical and subcortical structures.

Positron emission tomography (PET)^15^ imaging can isolate changes in neurotransmitter availability, neuronal metabolism, and molecular structural features within the central nervous system (e.g. quantification of amyloid or tau proteins utilizing molecular probes). A few PET neuroimaging studies have related regional alterations in metabolism, measured using fluorodeoxyglucose PET (FDG) and binding of a benzodiazepine/γ-aminobutyric acid (GABA) receptor ligand ([^11^C]-flumazenil (FMZ)), to behavioural outcomes in cross-sectional studies of subjects with TBI. These studies suggest that behavioural outcomes grade with overall recovery of cerebral metabolic rate^16,17^ and degree of gamma-aminobutyric acid (GABA_A_) binding.^18^ Specifically, they showed that reduction of GABA_A_ binding, measured using [^11^C]FMZ-PET, within the medial frontal cortices, anterior cingulate, and central thalamus correlates with executive dysfunction.^18^ A primary role for these regions in supporting recovery of consciousness and cognitive function following varying aetiologies of brain injury is proposed by the anterior forebrain mesocircuit (AFM) hypothesis.^19^ This hypothesis isolates the contribution of the central thalamus and its reciprocal connections with the frontal cortex and striatum (via cortico-striatopallidal-thalamic connections) to impaired arousal regulation and cognitive dysfunction following structural brain injuries. Prior studies support this hypothesis and several have related alteration of function within these same regions with levels of recovery up to *near* baseline performance of cognitive functions (reviewed in Giacino *et al.*,^20^ Thibault *et al.*,^21^ Edlow *et al.*,^22^ and below).

The GABA_A_ receptor is widely distributed throughout the brain and is in particularly high concentration within the cerebral cortex because of the numerous GABA-ergic inhibitory synapses.^23^ A reduction of GABA_A_ receptor binding measured by FMZ-PET has also been suggested as an in vivo marker of neuronal cell death.^18,24–31^ Decreased GABA_A_ receptor binding, as a measure of neuronal loss, has been verified with autoradiographic,^32^ direct ^33–35^ and indirect histopathological findings,^36^ and MRI imaging^31,36–38^ However, beyond neuronal loss, alterations in GABA_A_ receptor binding could also result from receptor dysfunction/reduced affinity as has been suggested by studies of isoflurane^39^ and tiagabine,^40^ both of which induced elevations in [11C]FMZ non-displaceable binding potential (BP_ND_). In rodents, an increased availability of GABA_A_ receptors^41^ has been associated with recovery following TBI.^42^ FMZ-PET binding alterations have also been correlated with cognitive deficits in ALS,^43^ multiple sclerosis,^31^ memory performance in Alzheimer’s disease^36^ and executive dysfunction following carotid artery disease.^38^

In this context, FMZ-PET could serve as an in-vivo marker of both permanent neuronal damage and/or functional recovery, especially as it relates to cognitive impairments as they change over time since injury. Preserved or mild reductions in binding might be an indication of intact structure, or of increased expression of GABA_A_ receptors in the remaining neurones ^40,44,45^ indicating a recruitable reserve for potential recovery. While prior studies suggest a relationship between FMZ-PET binding and cognitive function, they have not been tracked longitudinally during clinical recovery post-TBI. Changes in GABA availability can be expected to arise with shifts in large-scale network function and behavioural recovery that continue to evolve over long time periods following TBI.^19,46,47^ Here, we explore the use of FMZ-PET to measure and track changes in GABA_A_ availability following TBI, systematically, across brain regions and with a special emphasis on substructures identified as components of the AFM.^19^

## Materials and methods

### Study design

The study was designed to compare GABA_A_ receptor availability in adults with TBI to control individuals without TBI. We measured brain GABA_A_ receptor binding using PET imaging with the tracer [^11^C]FMZ. We included data from 9 (7 male) adults with TBI and 20 (12 male) adult controls. TBI participants underwent study activities at two timepoints: 3–6 months after injury (sub-acute, n = 9) and 11–20 months after injury (chronic, n = 7 of 9). Because the goal of the current study is to relate enduring cognitive impairments to the biological substrate changes following TBI, we focused on the sub-acute period for the first scan, to allow for sufficient time for acute medical issues to resolve. A subset of control individuals without TBI (n = 9) underwent a second scan 5–11 months after the initial visit.

### Participants and recruitment

TBI participants were recruited through inpatient rehabilitation units and trauma departments at large, urban academic medical centres. Control individuals were recruited through local advertisements. All study activities were approved by Weill Cornell Medicine’s Institutional Review Board.

All participants were required to meet the following criteria: (i) 18 years of age or above; (ii) English-speaking; (iii) capable of providing informed consent or a proxy/authorized agent available to provide informed consent; (iv) physically healthy and able to safely undergo PET imaging; (v) not currently taking any psychoactive or benzodiazepine drugs; (vi) not currently taking any medication for attention-deficit/hyperactivity disorder; (vii) no history of schizophrenia, drug, or alcohol abuse; (viii) no history of epilepsy, stroke, dementia, or serious medical illness by self-report; and (iv) not pregnant (for female participants).

Participants in the TBI group were required to have sustained a complicated mild (Glasgow Coma Scale^48^ score of 13–15 with evidence of intracranial lesion as verified on acute neuroimaging) or moderate-severe TBI (Glasgow Coma Scale score ≤ 12) within the last 6 months.

See Supplementary Tables 1 and 2 for further details.

### [^11^C]FMZ imaging data acquisition and processing

#### Radiopharmaceutical synthesis

The radioligand ethyl 8-fluoro-5,6-dihydro-5-[11C]methyl-6-oxo-4H-imidazo [1,5-a] [1,4] benzodiazepine-3-carboxylate, known as 11C-FMZ, was prepared by modifying previously described procedures.^49^ Briefly, a pre-mixed solution of desmethyl-FMZ (1 mg, 3.46 μmol) in anhydrous Dimethyl sulfoxide (0.35 mL) was added into the vial containing NaOH solution (1 M, 3 μL), and the vial was subjected to vortex to allow for well-mixing of the reaction mixture. After the collection of 11C-MeI, the vial was heated at 60°C for 1 min. It was then mixed and injected into reverse phase semi-prep high-pressure liquid chromatography for purification (Phenomenx Luna C18, 250 × 10 mm, 10 μ; 10 mL/min; 254 nm). The radioactive peak corresponding to 11C-FMZ was collected, diluted with H_2_O (60 mL), and passed through a pre-activated C18 plus cartridge (waters). The retained activity was eluted with ethanol (1 mL) and 0.9% saline (14 mL) through a 0.22 μ Millex GV filter into a sterile vial as final drug product. 11C-FMZ was prepared with an average radiochemical yield of 68.1 ± 12.5% (*n* = 43, decay corrected) and the average molar activity at the end of bombardment was 2016 ± 633 GBq/μmol (54.5 ± 17.1 Ci/μmoL), and the synthesis took 44 ± 3 min.

#### FMZ administration, image acquisition, and processing

A dose of 407–595 MBq (11–16mCi) of 11C-FMZ was administered intravenously to each subject. Tracer dosing is reported in Supplementary Table 2. A dynamic PET scan was performed over a period of 60 min beginning simultaneously with the tracer injection. PET data were acquired in 3D list mode with the same whole-body PET/CT scanner (mCT, Siemens/CTI, Knoxville, TN).The PET camera has a spatial resolution of ∼4 mm measured as the reconstructed full-width at half maximum of a point source in air. PET scans were corrected for photon absorption and scatter, using an in-line CT scanner set at 120 kV, a pitch of 1.5, and 30 mA. PET data were reconstructed in a 400 × 400 matrix with a voxel size of 1.082 × 1.082 × 2.025 mm^3^ using a zoom of 2.0 and an iterative + time of flight list-mode reconstruction algorithm provided by the manufacturer.^50^ FMZ-PET images were reconstructed into 22 frames (4 frames of 15 seconds (s) each, then 4 × 30 s, 3 × 60 s, 2 × 120 s, 8 × 300 s and 1 × 600 s).

#### PET data analysis

A time activity curve was extracted for each region of interest (ROI, see below). The simplified reference tissue model^51^ was used to calculate non-displaceable binding potential (BP_ND_) for FMZ-PET with pons acting as reference region (Supplementary Fig. 5). Summed PET images were coregistered to their corresponding MRI scans using rigid registration with mutual information. All kinetic analyses were performed using PMOD 3.5 (PMOD Technologies Ltd., Zurich Switzerland).^52^

### MRI data analysis

#### MRI data acquisition and processing

A 3T Siemens Prisma scanner with a 32-channel head coil was used to collect 3D T1-weighted sagittal MPRAGE anatomical images. All T1 volumes were processed using FreeSurfer 6.0^52^ to generate gray-matter regions of interest for subsequent PET analysis, as well as to estimate variability in gray-matter volume across the study population.To maximize signal-to-noise ratio and simplify statistical comparisons while preserving regional variation within the anterior forebrain mesocircuit (AFM), we combined the FreeSurfer gyral and subcortical ROIs and thalamic nuclei into larger groups. The six-lobe-based gyral groups were frontal, insular, polymodal, posteromedial cortex (PMC), temporal, and occipital groups. The five AFM cortical regions were anterior cingulate cortex (ACC), dorsolateral prefrontal cortex (dlPFC), ventrolateral prefrontal cortex (vlPFC), lateral parietal, and medial parietal. The seven subcortical groups were caudate, pallidum, putamen, anterior thalamus, ventral thalamus, posterior thalamus, and medial thalamus. See Supplementary Data and Supplementary Table 3 for further details.

### Cognitive measures

Subjects with TBI and uninjured control participants performed the Attention Network Test (ANT) paradigm,^5^ a computer-administered measure designed to examine the alerting, orienting, and executive attention networks. See Supplementary Data for details.

### Statistics

BP_ND_, region of interest (ROI) size, and attention-network scores were normalized and age-adjusted based on the control population. For each metric and each ROI, we computes the least squares linear effect of age for the uninjured population and then applied that slope and offset to remove the effect of age within the TBI group. This age-adjusted residual is then normalized by the mean and standard deviation of the age-adjusted residual from the uninjured population to produce an age-adjusted z-score. BP_ND_ measurements were simultaneously adjusted for both age and ROI size in this manner. For longitudinal changes, we computed the unadjusted difference across the two sessions before adjusting and normalizing. Group means were compared via an unpaired, two-tailed t test. Inter-metric relationships were examined using Pearson correlation, followed by a Student's *t*-test. For all analyses, α was set at 0.05, and two-tailed tests were used. All p-values reported are uncorrected for multiple comparisons. Data were analyzed and visualized using custom scripts in Matlab (version R2018b; www.mathworks.com).

### Data and materials availability

The study protocol will be available on request to the principal investigator. Data from this study are available from the corresponding author upon reasonable request.

## Results

### PET imaging with [^11^C]FMZ shows focal differences in GABA_A_ receptor availability among adults with TBI compared to controls during the subacute phase of recovery

**Figure 1:**
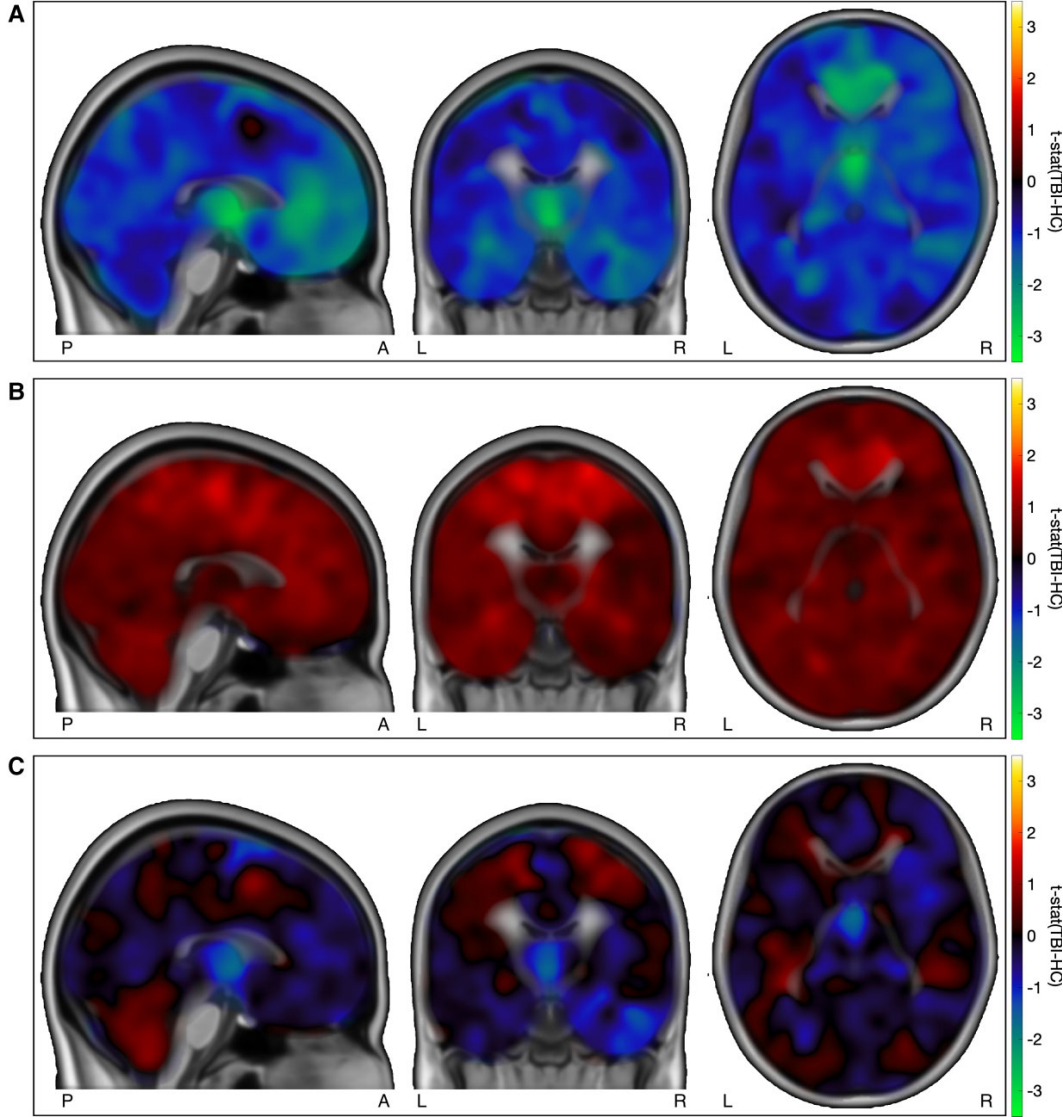
**Voxel-wise [^11^C]FMZ tracer BP_ND_ in individuals with TBI compared to controls.** HC: healthy controls. (**A**) Group differences in [^11^C]FMZ tracer BP_ND_ in individuals with TBI at sub-acute timepoint (*n* = 9) and control individuals without TBI (*n* = 20). Individual BP_ND_ volumes were nonlinearly registered to MNI and smoothed by 12 mm FWHM before adjusting for age and total intracranial volume, and computing a voxelwise unpaired *t*-test. Regions with values less than zero represent TBI_subacute_ < controls. Broad reduction of BP_ND_ appears across the subjects with TBI with predominant loss within medial frontal and thalamic regions. BPND values have been adjusted for age and total intracranial volume based on controls alone. (**B**) Longitudinal changes in [11C]FMZ tracer BP_ND_ in adults with TBI compared to between-scan changes in control individuals without TBI. Regions with values greater than zero represents longitudinal increases in subjects with TBI that were greater than longitudinal variability in controls. Regions with values less than zero represents longitudinal decreases in subjects with TBI that are greater than longitudinal variability in controls. Globally increased BP_ND_ across the paired scans is demonstrated in the subjects with TBI (controlled for by changes measured in control BP_ND_ over similar time difference). Changes in BP_ND_ have been adjusted for age and total intracranial volume differences between the groups. (**C**) Group differences in [^11^C]FMZ tracer BP_ND_ in individuals with TBI at chronic timepoint (*n* = 7) and control individuals without TBI (*n* = 20). Regions with values less than zero represent TBI_chronic_ < controls and regions with values greater than zero represent TBI_chronic_ > controls. A mixed pattern of regional changes in BP_ND_ is demonstrated with persistently lower thalamic BP_ND_ in the TBI group. BPND values have been adjusted for age and total intracranial volume.

Using the [^11^C]FMZ tracer for PET imaging, we found reductions in estimated [^11^C]FMZ binding potential (BP_ND_) in adults with TBI (*n* = 9) during the subacute phase of recovery compared to uninjured control individuals (*n* = 20) at their baseline assessment. In Fig. 1A, voxel-by-voxel comparisons highlighted BP_ND_ reductions in the medial frontal regions and thalamus.

To investigate regional differences, we first assessed changes in cortical thickness and subcortical volume following TBI (Supplementary Fig. 1A). We assessed the linear effect of age and total intracranial volume based on uninjured controls alone. After adjusting for these effects, we found differences in thalamic subcortical volume in individuals with TBI compared to uninjured controls (*P* < 0.05).

**Figure 2:**
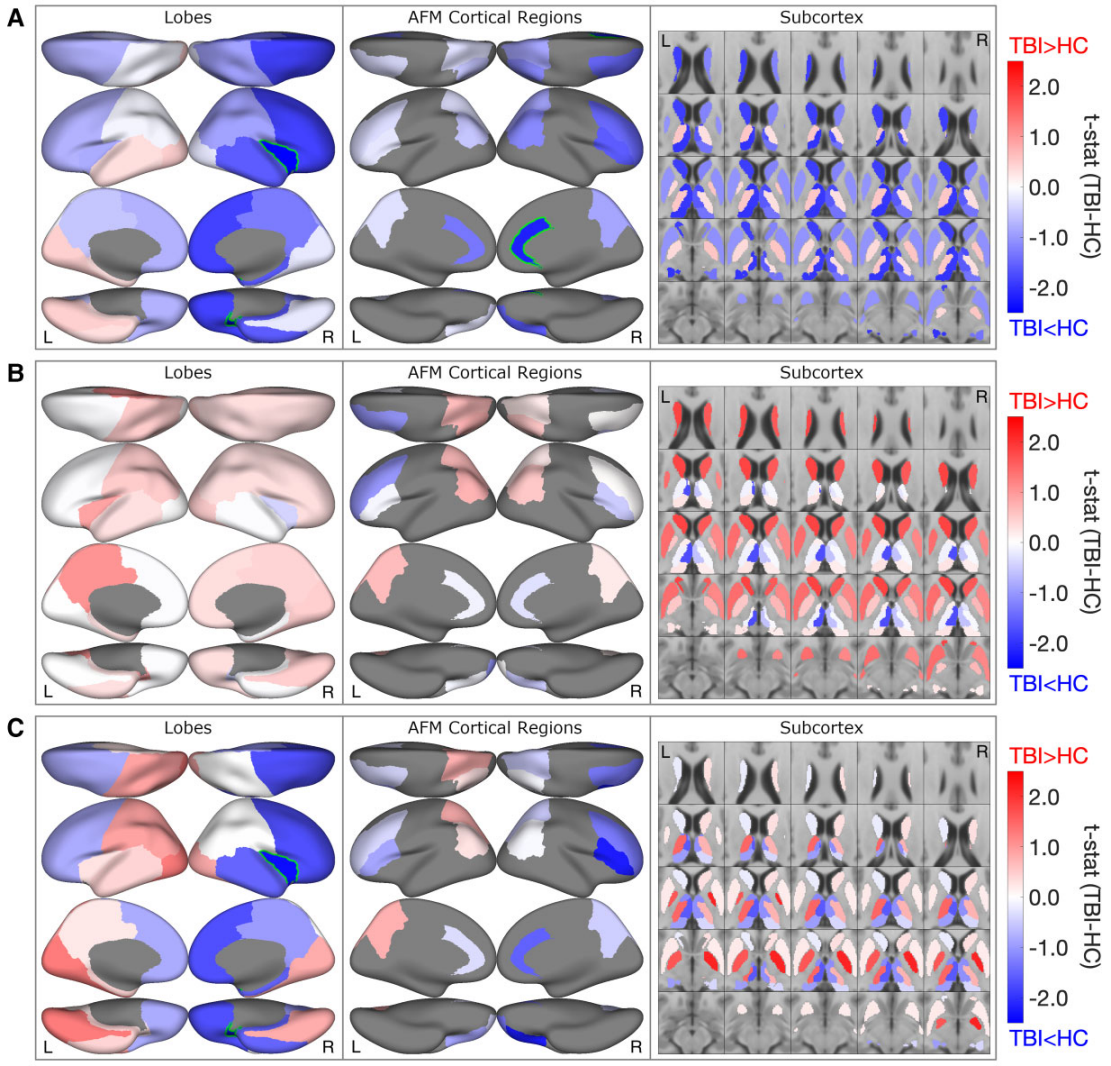
**Group-level regional [^11^C]FMZ tracer BP_ND_ in individuals with TBI compared to controls.** HC: healthy controls. (**A**) Group differences in [^11^C]FMZ tracer BP_ND_ in individuals with TBI at sub-acute timepoint (*n* = 9) and control individuals without TBI (*n* = 20). T-stat of the group differences. Outline: uncorrected *P* < 0.05. All regional values were z-scored after adjusting for age and cortical thickness/subcortical volume based on healthy controls. Regions with values less than zero represent TBI_subacute_ < controls and regions with values greater than zero represent TBI_subacute_ > controls. Reduced BP_ND_ is seen in the frontal lobes, striatum and posterior-medial thalami (R > L). (**B**) Longitudinal changes in [11C]FMZ tracer BP_ND_ in adults with TBI compared to between-scan changes in control individuals without TBI. Regions with values less than zero represent longitudinal decreases in subjects with TBI that are greater than longitudinal variability in controls. Regions with values greater than zero represent longitudinal increases in subjects with TBI that are greater than longitudinal variability in controls. Broad relative increases in BP_ND_ are seen across cortical regions in subjects with TBI excepting the left frontal lobe; subjects with TBI demonstrated increased BP_ND_ across bilateral caudate and putamen. Changes in BP_ND_ have been adjusted for age and cortical thickness/subcortical volume differences between the groups. (**C)** Group differences in [^11^C]FMZ tracer BP_ND_ in individuals with TBI at chronic timepoint (*n* = 7) and control individuals without TBI (*n* = 20). Regions with values less than zero represent TBI_subacute_ < controls and regions with values greater than zero represent TBI_subacute_ > controls. A heterogenous pattern of changes is noted with persistently lower bifrontal BP_ND_ and markedly increased bilateral pallidal BP_ND_ in subjects with TBI.

We then investigated regional differences in BP_ND_, within the lobes, following TBI. We again, first, establish the effect of age, total intracranial volume, cortical thickness and subcortical volume on BP_ND_ in uninjured controls (see Supplementary Fig. 2 for four example regions). We note a decrease in BP_ND_ with age (right/left gray matter; Pearson’s *r* = −0.39/−0.28; *P* = 0.09/0.23) (Supplementary Table 4A). We also note a decrease in BP_ND_ with larger intracranial volume (right/left gray matter; Pearson’s *r* = −0.41/−0.36; *P* = 0.09/0.14). In Fig. 2A and Supplementary Fig. 3A, regional analyses of BP_ND_ in subjects with TBI, corroborated the voxel-based findings (Fig. 1A) but, additionally, highlighted a marked asymmetry in the BP_ND_ reductions; this is after adjusting for age and cortical thickness/subcortical volume (trends consistent in unadjusted values, see Supplementary Fig. 4). Across the group, the right cortical regions had larger reductions than left (right/left: *t*_29_ = −1.77/−0.36, *P* = 0.10/0.72). These reductions were most pronounced in the right insula (*t*_29_ = −2.66, *P* = 0.02) and right frontal lobes (*t*_29_ = −1.80, *P* = 0.09). At the lobar level, group (TBI versus controls) differences in cortical thickness and subcortical volume show a similar spatial pattern to those seen in the group differences in cortical BP_ND_ (Pearson’s *r* = 0.69, *P* = 0.013); this is after accounting for age and intracranial volume (see Supplementary Figs 6 and 7).

Lastly, we investigated BP_ND_ within the anterior forebrain mesocircuit (AFM) regions^19^ (Fig. 2A and Supplementary Table 3) and note that reduced BP_ND_ following TBI was most pronounced in the right anterior cingulate (ACC, *t*_29_ = −2.10; *P* = 0.05), right vlPFC (*t*_29_= −1.63; *P* = 0.13), and right dlPFC (*t*_29_ = −1.50; *P* = 0.16). Reduced BP_ND_ was also seen in the thalamic parcellations: anterior (right *t*_29_ = −1.92; *P* = 0.07), posterior (right *t*_29_ = −2.00; *P* = 0.06), and medial (right *t*_29_ = −1.95; *P* = 0.08). Similarly, reduced BP_ND_ is noted in the right caudate (*t*_29_ = −1.90; *P* = 0.07). The spatial patterns of the group differences in BP_ND,_ within the AFM regions, were not significantly correlated with the group differences in cortical thickness and subcortical volume (see Supplementary Fig. 6).

All BP_ND_ derivations are referenced to the pons,^53^ similar time-activity curves in both groups confirm this choice (Supplementary Fig. 5). We also investigated image derived input function using a carotid artery ROI, but the BP_ND_ results were noisier even in uninjured controls.^50^

### Longitudinal PET imaging with [^11^C]FMZ shows focal changes in GABA_A_ receptor availability among adults with TBI

We assessed changes in [^11^C]FMZ BP_ND_ over the first year of recovery following TBI and compared this to changes over a similar time-frame in uninjured controls. In seven subjects with TBI, the first, sub-acute scans were 3–6 months after injury and the second, chronic scans were 11–20 months after injury. Across the cohort, the median between-scan time was 9 months (range 7–13 months). Of the 20 uninjured controls, 9 were scanned twice; the median between-scan time was 5.7 months (range 5–11 months).

First, we assessed changes in cortical thickness and subcortical volume between the two scans in subjects with TBI (Supplementary Fig. 1B; Supplementary Fig. 6); we account for the effect of age and intracranial volume in this analysis. We find an overall decrease in cortical thickness, especially in the right lateral-parietal region (*t*_16_ :−2.31; *P* = 0.05) and postero-medial cortex (right/left *t*_16_ : −2.88/−2.68; *P* = 0.01/*P* = 0.02).

We then assessed for changes in BP_ND_ between the two scans. In subjects with TBI, we find widespread increases in BP_ND_ that exceeded longitudinal variability seen in uninjured controls (Figs. 1B and 2B; Supplementary Fig. 4),even after we account for the effect of age and differences in cortical thickness and subcortical volume between the groups. The largest increases in BP_ND_ occurred in the subcortical regions of caudate (right/left *t*_16_: 1.62/1.83; *P* = 0.14/*P* = 0.11) and putamen (right/left *t*_16_: 1.17/1.41; *P* = 0.27/*P* = 0.19) (Supplementary Table 4B). While increases are seen in several of the cortical regions, particularly in the left hemisphere, very small changes or further reductions in BP_ND_ are noted in the frontal cortex.

In summary, limited focal increases in GABA_A_ receptor availability occur within the frontal cortex during the first year of recovery following TBI. In addition, subcortical GABA_A_ receptor availability shows substantial increases over the same time period when compared with uninjured controls.

### PET imaging with [^11^C]FMZ shows focal increases and reductions in GABA_A_ receptor availability among adults with TBI during the chronic phase of recovery compared to uninjured controls

At the chronic scan in subjects with TBI, we again assess differences in cortical thickness and subcortical volume compared to the baseline scan in uninjured controls; this is after accounting for age and intracranial volume (Supplementary Fig. 1C; Supplementary Fig. 6). We note an increase in cortical thickness in the left insula (*t*_16_ : 2.52; *P* = 0.02) and left occipital cortex (*t*_16_ : 2.22; *P* = 0.05). We also note a decrease in volume in the left anterior (*t*_16_:−2.37; *P* = 0.03) and ventral thalamus (*t*_16_ :−2.28; *P* = 0.04).

In subjects with TBI, BP_ND_ within the pallidum exceeds levels seen in the baseline scan of uninjured controls (right/left t16: 1.65/2.13; P = 0.14/P = 0.06), Figs. 1 and 2C; Supplementary Fig. 4, (Supplementary Table 4C); this is after accounting for age, cortical thickness, and subcortical volume. Conversely, in the frontal cortex, BP_ND_ remained below levels seen in uninjured controls, particularly in the right insula (*t*_16_ : −2.37; *P* = 0.03) and right vlPFC (*t*_16_ : −2.21; *P* = 0.05).

Despite the widespread increases in GABA_A_ receptor availability during the first year of recovery, within the frontal cortex, chronically, BP_ND_ remain below levels seen in uninjured controls.

### Longitudinal improvement of [^11^C]FMZ binding potential in subjects with TBI is related to improvement in executive attention.

We measured executive attention in subjects with TBI in conjunction with coregistered PET and MRI scans at 3–6 months post-injury and 11–20 months post-injury. The Attention Network Test (ANT)^5,54^ is a computerized, response-time dependent measure of three aspects or ‘networks’ of attentional functioning, including alerting attention, orienting attention, and executive attention (conflict resolution and inhibition).^5,9^ For the ANT, we calculated age-corrected z-scores from a healthy control dataset (existing laboratory dataset, including the 20 healthy subjects included in this study) of 66 individuals ranging from age 22 to 75. We tested the correlation of longitudinal change in BP_ND_ with change in executive attention as measured by the ANT (*n* = 6, after removing one subject outlier) (Supplementary Fig. 8). Broad increases in BP_ND_, over most of the cerebrum, correlated with an improvement in executive attention (decrease in ANT executive network score). Conversely, a longitudinal increase in BP_ND_ within the caudate, putamen, pallidum and posterior thalamus (see also Figs. 1C and 2C) showed negative correlation with these behavioural improvements.

## Discussion

Longitudinal flumazenil (FMZ) PET measurements of GABA_A_ receptor availability in the brains of adults with TBI revealed broad and consistent changes following injury when compared to adults without TBI. We find that GABA_A_ receptor availability decreases across cerebral structures following TBI; the most predominant reductions are within the frontal lobes, striatum, and posterior-medial thalami at the subacute (3–6 months post) time point. Within the frontal cortices, the reductions showed significant differences from uninjured controls in the right insula and ACC. Comparison of sub-acute and chronic (11–20 months post) BP_ND_ demonstrated widespread changes in the GABA_A_ receptor availability characterized by global increases in BP_ND_. Despite these broad increases in BP_ND_, subjects with TBI showed a heterogeneous pattern of changes in BP_ND_ when compared to uninjured controls. At the chronic time point, subjects with TBI demonstrate persistently lower BP_ND_ in the thalami and bi-frontal cortices and bilaterally increased pallidal BP_ND_. In subjects with TBI, improvements in executive function captured in the performance of the Attention Network Task (ANT) correlated with increases in BP_ND_ across bilateral fronto-parietal cortical regions and the anterior-lateral aspects of the thalami. In the aggregate, FMZ PET measurements of GABA_A_ receptor availability demonstrated a clear sensitivity to changes in brain structure and function after TBI and strong evidence of evolving changes over the time course of the first year of recovery. The specific observations of persistent bi-frontal lobe depression and bilateral pallidal elevation of BP_ND_ are notable as they verify a strong prediction of the anterior forebrain mesocircuit (AFM) model for recovery from TBI, as discussed below. These data support prior observations in cross-sectional studies of FMZ PET in subjects with TBI and greatly extend the potential for this tool to elucidate specific cellular mechanisms underlying the recovery process and persistent cognitive deficits. Our findings provide a foundation for the future use of FMZ PET to track recovery in TBI and measure response to possible therapeutics. Specifically, we anticipate that patterns of GABA_A_ availability will track those observed here in spontaneous recovery graded by improved performance on tests of executive function and show causal linkage to our interventions in future study designs. In this way, FMZ PET will allow us to correlate behavioural changes induced by the interventions to obtain unparalleled precision of circuit level changes.

### Acute TBI globally depresses GABAA receptor availability as measured by [^11^C]FMZ PET

The observations of focal reductions in BP_ND_ within the frontal lobes, anterior cingulate gyrus, and the thalamus following TBI, are consistent with prior cross-sectional [^11^C]FMZ studies.^18,30,55–57^ Similarly, our findings of inter-individual variability in the BP_ND_ are consistent with previous reports.^18,30,55^ The notable differences between our study and these previous reports are as follows. Our sample spans a fuller range of TBI injury severity from complicated mild to severe compared to previous studies,^18^ and patients in our study were not selected a priori by degree of cognitive impairment or cortical damage. We restricted our first scan to 3–6 months post injury and our second scan to 11–20 months post injury. In contrast, the single scans, in the previous cross-sectional studies, varied widely in time since injury: 2–17 months,^55^ > 6 months,^57^ >1 year^30^ and 12–228 months.^18^ Our healthy control sample (*n* = 20, age range: 22–65 years) also differed from previous studies which limited enrolment to those under age 30^18^ or 40^30^ years old. Thus, the effect of age on BP_ND_ in healthy controls was not explored in these prior studies, nor, adjusted for in the TBI findings. Our findings of an effect of age on BP_ND_ in healthy controls has, however, been reported before in [^11^C]FMZ PET studies of epilepsy.^58^ The other studies also did not account for atrophy changes following TBI, as we have done for all our BP_ND_ findings. Despite these various methodological differences with prior studies, our confirmation of a dominant depression of GABA_A_ receptor availability following TBI and marked loss of signal within the medial frontal lobes and thalamus supports the generalizability of this result seen across prior FMZ PET studies in TBI.

The broad reductions in BP_ND_ suggest a mix of underlying factors. Reductions in GABA_A_ receptor availability has been proposed as a marker of neuronal cell death as the GABA_A_ receptor is the most abundantly occurring neuronal receptor.^36^ However, reduced binding could also reflect a reduced population of GABA_A_ receptors secondary to changes in synaptic activity (e.g. GABA_A_ receptors are known to rapidly internalize under conditions of status epilepticus^59,60^ and undergo altered trafficking and receptor properties leading to dysfunction^61^ in the setting of TBI and other neurological disorders).^62^

Our FMZ-PET results are also comparable to prior cross-sectional [18F] fluorodeoxyglucose PET studies of TBI patients which demonstrate graded thalamic and medial frontal hypometabolism that correlates with behavioural outcomes.^16,17,63–65^ Nakayama *et al.*^63^ found that diffuse traumatic brain injuries showed a consistent hypometabolism in medial bifrontal, cingulate gyrus and thalamus that graded with severity of behavioural impairment across patients with moderate cognitive disability through vegetative state outcomes. Garcia-Panach *et al.*^17^ found similar changes in FDG-PET patterns of cerebral metabolism and TBI outcomes but specifically identified decreased medial frontal and thalamic metabolism in patients who emerged from post-traumatic amnesia and controls, similar to our observations. Nakashima *et al.*^65^ reported on hypometabolism within the cingulate gyrus in subjects with TBI with neuropsychologic deficits. Other studies have isolated reduced thalamic metabolism with TBI in early^66^ and, more comparably, late time points after initial TBI.^64^ One study specifically correlated reductions in the anterior cingulate FDG-PET metabolic signal with neuropsychological deficits following diffuse axonal injuries.^16^ These prior findings of selectively reduced cerebral metabolism in the same regions in which BP_ND_ is reduced suggest an important functional component to the loss of GABA_A_ receptor availability particularly within medial frontal and thalamic regions as seen in Fig. 1A.

### [^11^C]FMZ PET reveals spatially specific changes in GABAA receptor availability from the initial subacute period to 1 year following TBI

Longitudinal measurements revealed significant changes in the BP_ND_ between the sub-acute time point and ∼1 year time-point that exceed the measured longitudinal variability in our controls across a similar time interval. No prior studies of subjects with TBI have evaluated interval changes in [^11^C]FMZ PET measured GABA_A_ receptor availability; thus, these findings provide the first evidence that FMZ PET is sensitive to evolving changes in the expression of the GABA_A_ receptor on neurones across the cerebrum following TBI. Notably, while frontal cortical regions showed persistent and significant loss of GABA_A_ receptor availability, a specific pattern of subcortical changes within the striatum and pallidum emerged. In the striatum, marked increases from initially decreased BP_ND,_ are observed (Fig. 1B). Changes within the pallidum over the same time-frame results in ‘greater-than-normal’ BP_ND_ at chronic assessment. This novel finding could represent a functional increase in local inhibitory activity resulting from functional or structural deafferentation of excitatory descending inputs from the frontal cortex and/or central thalamus.^21^ In this context, our findings of longitudinal changes in GABA_A_ receptor availability may thus reflect (i) alterations in expression and affinity of GABA_A_ within preserved or remaining neuronal structure,^44,45^ and/or (ii) evidence of receptor plasticity.^42^ Previous studies have reported a return to baseline^67^ in glutamate signalling by 6 months post concussions and suggested chronic excitatory-inhibitory signalling imbalances (glutamate-GABA) following mild TBI.^68,69^ However, no studies provide comparable timelines and methodologies to directly compare our findings.

### Correlation of changes in cognition and concomitant changes in [^11^C]FMZ PET measured BPND within the first year following TBI

We demonstrate a significant correlation of increased GABA_A_ receptor availability with improved ANT executive attention score. The ANT executive score is a measure of inhibition and conflict resolution.^9^ These findings can be compared to a previous study^18^ of eight severe TBI patients—preselected by behavioural impairment and limited cortical damage; in this group, a graded FMZ binding reduction in the right thalamus and left medial frontal gyrus, during the chronic phase of recovery (12–228 months), was associated with lower general cognitive function (full-scale IQ). Another study^57^ reported that the majority of patients with memory impairment (*n* = 6 of 9) had low binding potential in the temporal lobe. In our results, we show an overall global increase in BP_ND_ within cortical structures and thalami relates to an improvement in executive attention behaviour. The relationship between GABA receptor availability and executive attention is particularly relevant to recovery given the strong correlation between executive functions and daily activities.^70,71^

### Changes in [^11^C]FMZ PET BPND over the first year following TBI are consistent with the AFM model of recovery

Collectively, the changes seen here in GABA_A_ binding reflected in the [^11^C]FMZ PET BP_ND_ are strongly consistent with the AFM model of recovery of consciousness and brain function following varying aetiologies of brain injury.^19,72^ The mesocircuit model begins with a single assumption that multi-focal brain injuries result in a broad reduction of background synaptic activity across the corticothalamic system. This emerges as a consequence of widespread deafferentation and is amplified by the disfacilitation of central thalamic regions which act as a critical hub in the forebrain arousal regulation system.^19,72^ The model proposes that marked reduction in frontal cortical and striatal function arise via functional and structural deafferentation of central thalamic projections to these structures. These changes are proposed to be secondary to: (i) the strong central thalamic anatomical projections to these structures^73^ and (ii) the effects of chained inhibition within the cortico-striato-pallidal-thalamocortical loop systems.^74^ Down-regulation of striatal activity is predicted to arise as a consequence of reduced thalamostriatal/thalamocortical and cortico-striatal outflow in turn leading to disinhibition of the globus pallidus interna that results in additional direct inhibition of thalamic efferent activity. Thus, the mesocircuit model predicts the specific pattern seen in the subacute period here of reduced GABA_A_ binding across the frontal lobes and striatum (Figs. 1 and 2A) and in particular the marked increase in local pallidal GABA_A_ binding consistent with the emergence of bilateral increases in pallidal activity exceeding control levels most prominently at the second time point (Figs. 1 and 2C). Further, the accentuated metabolic depression within the medial frontal regions and thalami (Fig. 1A) correlate with several other studies demonstrating this prediction of the model^16,17^ (see Edlow *et al.*,^22^ Thibault *et al.*^21^ for review). Our findings can specifically be compared to a recent study^75^ in which secondary thalamic pathology was strongly correlated with outcomes following moderate to severe brain injury patients following TBI. In addition, a correlation of recovery of executive function with broad increases in [^11^C]FMZ PET BP_ND_ across cerebral structures (Supplementary Fig. 8) is also consistent with the mesocircuit model.

Fridman *et al.*^72^ examined the ratio of local metabolic activity measured using [18F]fluorodeoxyglucose PET within the globus pallidum and central thalamus (GP/CT) in a cross-sectional study of patients with varying levels of recovery from severe brain injury. The GP/CT ratio showed an inverse correlation with increasing recovery of consciousness and restoration of higher-brain brain function. These observations are consistent with the findings here of chronically reduced frontal lobe and elevated GP [^11^C]FMZ PET BP_ND_ as reduced cortico-striatal and thalamo-striatal activity would be predicted to correlate with release of local pallidal neuronal firing.^19,74^ The increase in pallidal GABA_A_ binding likely reflects local circuit inhibitory activity; prior studies have demonstrated that microinfusion of zolpidem (a GABA_A_ alpha 1 selective positive allosteric modulator) within the pallidum can suppress such activity.^76^ Moreover, human globus pallidus interna is enriched with the GABA_A_ alpha-1 subunit.^77^ Targeting of overactive pallidal GABA_A_ activity in the setting of marked functional deafferentation of the frontal cortex and striatum has been proposed as a key component of paradoxical behavioural improvements seen in a minority^78^ of patients with disorders of consciousness following structural brain injuries with administration of zolpidem.^79^

While limited by multiple comparisons made in a small sample, our findings that right ACC showed significant change are also notable in the context of the mesocircuit model. The ACC has widespread projections across Layer 1 of frontal and pre-frontal cortices^80^ and is thus positioned to integrate loss of activity and connectivity across these structures. In the context of the role of the projections from the central thalamus to the fronto-striatal regions,^73^ these anatomical specialization can be envisioned to act as a ‘repeater circuit’ and amplify effects of arousal regulation through pre-frontal and frontal regions. Underactivation of these regions at baseline might provide the anatomical basis for reduced readiness to deploy executive attention observed in TBI patients with moderate to severe brain injuries.^9^ In this context, recent observations that patients with chronic cognitive impairments (Glasgow Outcome Scale-Extended^81^ scores 5–7) show improvement with central thalamic deep brain stimulation (DBS) with driving of the central thalamus into frontal cortex and striatum particularly in the Trail Making Test-B performance^82^ suggest that the reduced frontal GABA_A_ activity may provide a marker of a recruitable reserve.

Overall, the changes seen of globally increased GABA binding in Fig. 1B are consistent with increased arousal and relative reafferentation of the cortex and striatum arising over the first year following TBI; such a gradual shift in forebrain arousal and change in baseline activity would be consistent with the well-characterized improvements in behavioural recovery that continue over 1, 2, 5, and even 15 years following TBI.^83,84^ Within neocortical neurons, increased arousal is dominated by inhibitory currents^85,86^ and controlled by increased overall excitatory neurotransmission via long-range projections across the cerebrum. Increased arousal levels are associated with increased resting metabolism^87^ and would be predicted to increase the number of GABA receptors on neocortical neurons over time. GABA_A_ receptors mediate fast responsive changes in cortical tone^88^ and play a key role in the strong background inhibitory currents associated with awake ‘activated’ states of the cortex.^85^ Thus, we propose that the increases observed across all brain regions between the subacute and chronic (∼1 year) time points of measurement here (Fig. 1B) are consistent with the FMZ signal tracking increased arousal and restoration of synaptic background activity. Importantly, this activity does not return to control levels across the frontal lobe, a finding that is consistent with a wide literature of predominantly frontal deficits that endure and impair vocational reentry, continuing education and social re- engagement following moderate to severe TBI.^89,90^

### Limitations

In the aggregate, our observations suggest the future use of [^11^C]FMZ as a measure to track spontaneous recovery or influence of pharmacological agents^91^ but also as an outcome measure of dynamic changes induced by electrical brain stimulation over multiple time scales. As such, PET more generally has tremendous advantages in not being sensitive to the artefacts produced by deep brain stimulation (DBS) electrodes in magnetic resonance based techniques and with the use of additional ligands^92^ allows a detailed molecular neuroimaging of either spontaneous recovery or therapeutic responses. The primary limitation of this study, similar to other existing [^11^C]FMZ PET studies^18,30,56,57^ of subjects with TBI, is its small sample size along with the heterogeneity of injury severity and wide range of ages. The range of cognitive impairments, though not selected for apriori, is relatively comparable. These constraints limit the generalizability of our results; nonetheless, we find a strong inter consistency of changes across cortical regions and subcortical structure comparable with those obtained using entirely independent methods in subjects with TBI as noted above. While we have detailed cognitive assessments in our subjects with TBI, statistical considerations of a small sample prevent finer stratification of our BP_ND_ results by cognitive impairment. We cannot draw strong conclusions about individual recovery trajectories and changes in GABA_A_ receptor availability as we have very few longitudinally repeated measurements. The limited GABA_A_ increases in the frontal cortex may be influenced by heterogeneity of injury severity and recovery in our sample. In a larger sample, we might expect to relate these factors to specific patterns of changes in GABA_A_. A larger cohort of healthy controls across a wider age range, and with test-retests across multiple timelines, would help to establish more precise quantitative expectations in control subjects. Moreover, GABA_A_ receptors are highly dynamic over time and recent studies have shown marked alteration of subunit types can arise in response to activity changes in network state (e.g. during status epilepticus^59,60^ or propofol anaesthesia.^93^ GABA receptors are rapidly trafficked to the neuronal membrane and can become dysfunctional in neurological disorders.^62^ Our measurements do not account for changes in subunit composition without alteration in nominal density of receptors nor the functional normality of the GABA receptors reflected in the binding we measure. We did not perform traditional partial volume correction (PVC) and instead chose to adjust for region size, as traditional PVC methods like geometric transfer matrix^94^ correction may increase noise and assume homogeneous values across regions. Our approach, however, does not take account of the spatially and temporally variant contrast in radioactivity concentration that induces the partial volume error. Future studies will compare PVC methods. Lastly, the use of a single ligand, specific to GABA_A_ receptors (and largely sensitive to the ɑ1 subunit given its 10-fold increased concentration^95^) limits our ability to assess the full range of changes in GABA receptor availability in our subjects and possible functional and structural alterations signalled by changes in this receptor type following TBI.

## Supplementary Material

fcac159_Supplementary_Data

## Abbreviations

ACC: anterior cingulate cortex
ADHD: attention-deficit/hyperactivity disorder
AFM: anterior forebrain mesocircuit
ALS: amyotrophic lateral sclerosis
ANT: Attention Network Test
BP_ND_: non-displaceable binding potential
DBS: deep brain stimulation
dlPFC: dorsolateral prefrontal cortex
FMZ: flumazenil
PMC: posteromedial cortex
PVC: partial volume correction
ROI: region of interest
SRTM: simplified reference tissue model
TBI: traumatic brain injury
TMT-B: Trail Making Test-B
vlPFC: ventrolateral prefrontal cortex
