# Supplementary material for: Longitudinal alterations in gamma-aminobutyric acid (GABAA) receptor availability over ∼ 1 year following traumatic brain injury
Source: Brain Commun. 2022 Jun 15;4(4):fcac159. doi: 10.1093/braincomms/fcac159 (PMC9253887; doi:10.1093/braincomms/fcac159)
Supplement: fcac159_Supplementary_Data [file fcac159_supplementary_data.docx]

# SUPPLEMENTARY MATERIALS

**Supplementary data**

MRI data acquisition and processing

Cognitive measures

**Supplementary figures**

Fig. S1. Group-level cortical thickness-subcortical volume in individuals with TBI compared to HCs

Fig S2. Relationship of healthy control (HC) binding potential (BP_ND_) with age, ROI size and total intracranial volume (ICV)

Fig. S3. Distribution of regional [11C]flumazenil tracer BP_ND_ in individuals with TBI compared to HCs.

Fig. S4. Distribution of regional [11C]flumazenil tracer BP_ND_ in individuals with TBI compared to HCs (not adjusted for age).

Fig S5. Standardized uptake value (SUV) time activity curves for healthy controls and subjects with TBI.

Fig. S6. Distribution of cortical thickness-subcortical volume in individuals with TBI compared to baseline scan in HCs

Fig. S7. Distribution of BP_ND_ in individuals with TBI compared to baseline scan in HCs

Fig. S8. Correlation of change in BP_ND_ in subjects with TBI (n=6) with change in ANT executive behavior z-score

**Supplementary tables**

Table S1. Participant demographic data for the subjects with TBI.

Table S2. Participant demographic data for the TBI and healthy control cohorts.

Table S3. Region of interest definitions.

Table S4. T-tests for group level differences in [11C]flumazenil tracer BP_ND_ between subjects with TBI compared to HC subjects.

**Supplementary references**

**Supplementary data**

###

### **MRI data acquisition and processing**

### A 3T Siemens Prisma scanner with a 32-channel head coil was used to collect 3D T1-weighted sagittal MPRAGE anatomical images. For the first 10 healthy controls and 2 TBI patients, these images were 1.2 x 1.2 x 1.2mm. For the remaining 10 healthy controls and 7 TBI patients, the anatomical scans were 0.8 x 0.8 x 0.8mm for visit 1 and visit 2. One subject in each group (uninjured controls and subjects with TBI) had PET and MRI scans 5 weeks apart. Five uninjured controls had scans within one week. All others had both scans on the same day. All T1 volumes were processed using FreeSurfer 6.0[^1^](https://www.zotero.org/google-docs/?X14laS) to generate gray-matter regions of interest for subsequent PET analysis, as well as to estimate variability in gray-matter volume across the study population. We used the probabilistic thalamic nuclei atlas from FreeSurfer 7.0 to generate 50 thalamic sub-parcels for each subject.[^2^](https://www.zotero.org/google-docs/?0rFSju) 44 out of these 50 sub-parcels were then merged into 8 left and right anterior, ventral, posterior, and medial thalamic groups according to the groupings in Supplementary Table 3.

The longitudinal FreeSurfer pipeline was used for the 9 healthy controls and 7 TBI patients with multiple visits, in order to robustly investigate changes in gray matter volume over time. No significant changes over time were observed, and for maximum stability, the parcellations from each subject’s first visit were used when analyzing PET data from both visits. To account for any residual effect of small changes in ROI size, the ROI sizes from each visit were used as covariates during statistical analyses. ROI size comparisons used total volume (mm^3^) for subcortical regions, and average cortical thickness (mm) for cortical regions.To maximize signal-to-noise ratio and simplify statistical comparisons while preserving regional variation within the anterior forebrain mesocircuit, we combined the FreeSurfer gyral and subcortical ROIs and thalamic nuclei into larger groups. The 6 lobe-based gyral groups were frontal, insular, polymodal, posteromedial cortex (PMC), temporal, and occipital groups. The 5 anterior forebrain mesocircuit cortical regions were anterior cingulate (ACC), dorsolateral prefrontal cortex (dlPFC), ventrolateral prefrontal cortex (vlPFC), lateral parietal, and medial parietal. The 7 subcortical groups were caudate, pallidum, putamen, anterior thalamus, ventral thalamus, posterior thalamus, and medial thalamus. See Supplementary Table 4 for details.

## Cognitive measures

Subjects with TBI and uninjured control participants performed the ANT paradigm[^3^](https://www.zotero.org/google-docs/?12xSXL), a computer-administered measure designed to examine the alerting, orienting, and executive attention networks. Age-normed z-scores were calculated for each of the three ANT networks and overall response time (RT) by using an unpublished dataset of 66 healthy adult ANT scores. In that dataset, we computed regression equations to predict ANT network scores and RT from age (i.e., ANT predicted score = y + B * age). We calculated z-scores for subjects with TBI by subtracting their obtained ANT score from the age-predicted score and dividing by standard deviation of the healthy adult database. Signs were flipped for ANT Executive and RT so that negative values always denote performance worse than healthy controls.

**Supplementary figures**


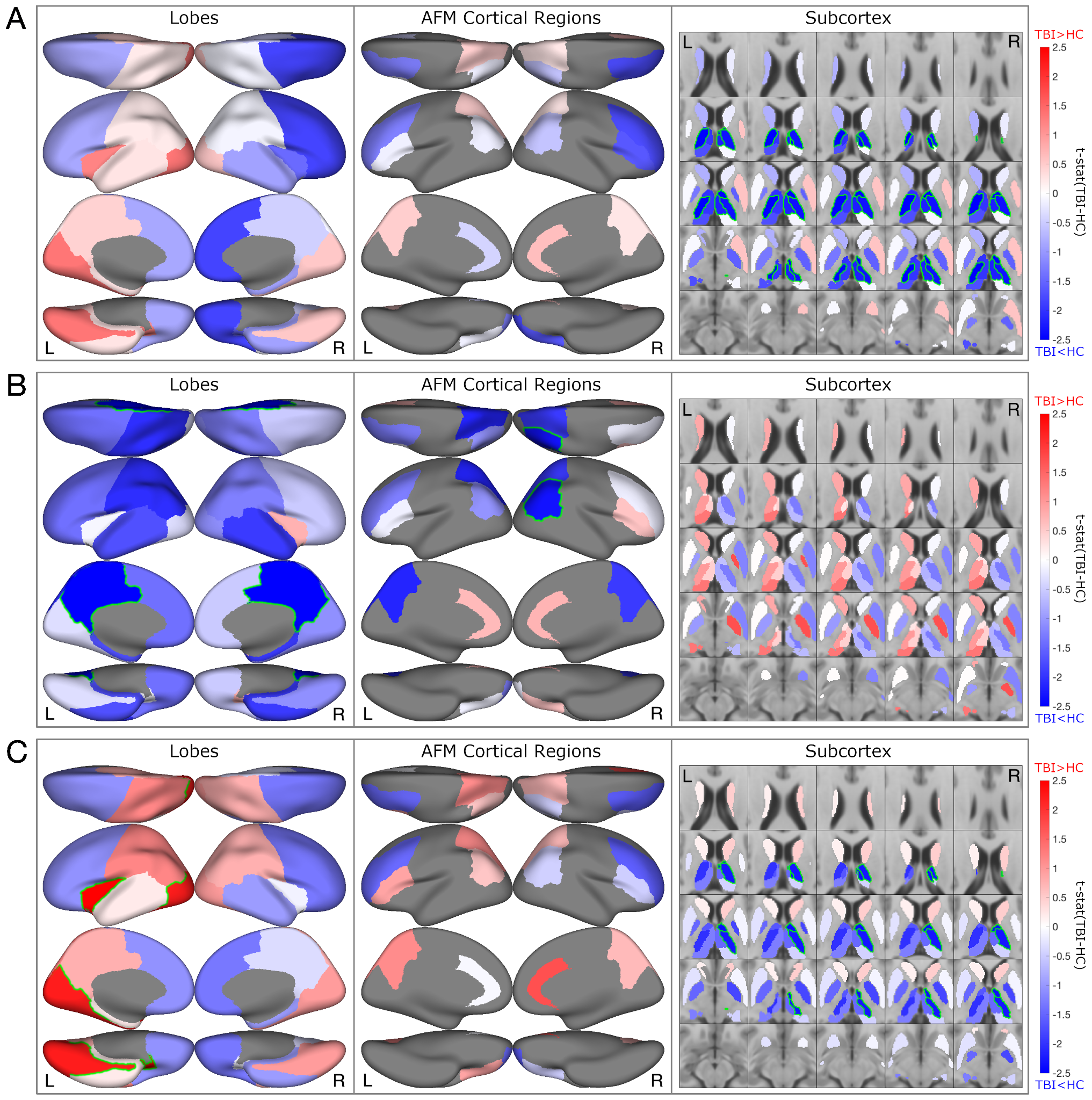


**Supplementary Figure 1.** **Group-level** **cortical thickness-subcortical volume in individuals with TBI compared to HCs**. **A:** Group differences in cortical thickness-subcortical volume in individuals with TBI at sub-acute timepoint (n=9) and control individuals without TBI (n=20). T-stat of the group differences. Green outline: uncorrected p<0.05. All regional values were z-scored after adjusting for age and total intracranial volume. Blue regions represent TBI_subacute_ < HC and pink regions represent TBI_subacute_ > HC. **B:** Group differences in cortical thickness-subcortical volume in individuals with TBI compared to between-scan changes in control individuals without TBI. Red-Yellow shading represents longitudinal increases in subjects with TBI that were greater than longitudinal variability in those without TBI. Red-pink shading represents longitudinal increases in subjects with TBI that are greater than longitudinal variability in those without TBI. Blue shading represents longitudinal decreases in subjects with TBI that are greater than longitudinal variability in those without TBI. Results have been adjusted for age and total intracranial volume. **C:** Group differences in cortical thickness/subcortical volume in individuals with TBI at chronic timepoint (n=7) and control individuals without TBI (n=20). Blue regions represent TBI_chronic_ < HC and pink regions represent TBI_chronic_ > HC.


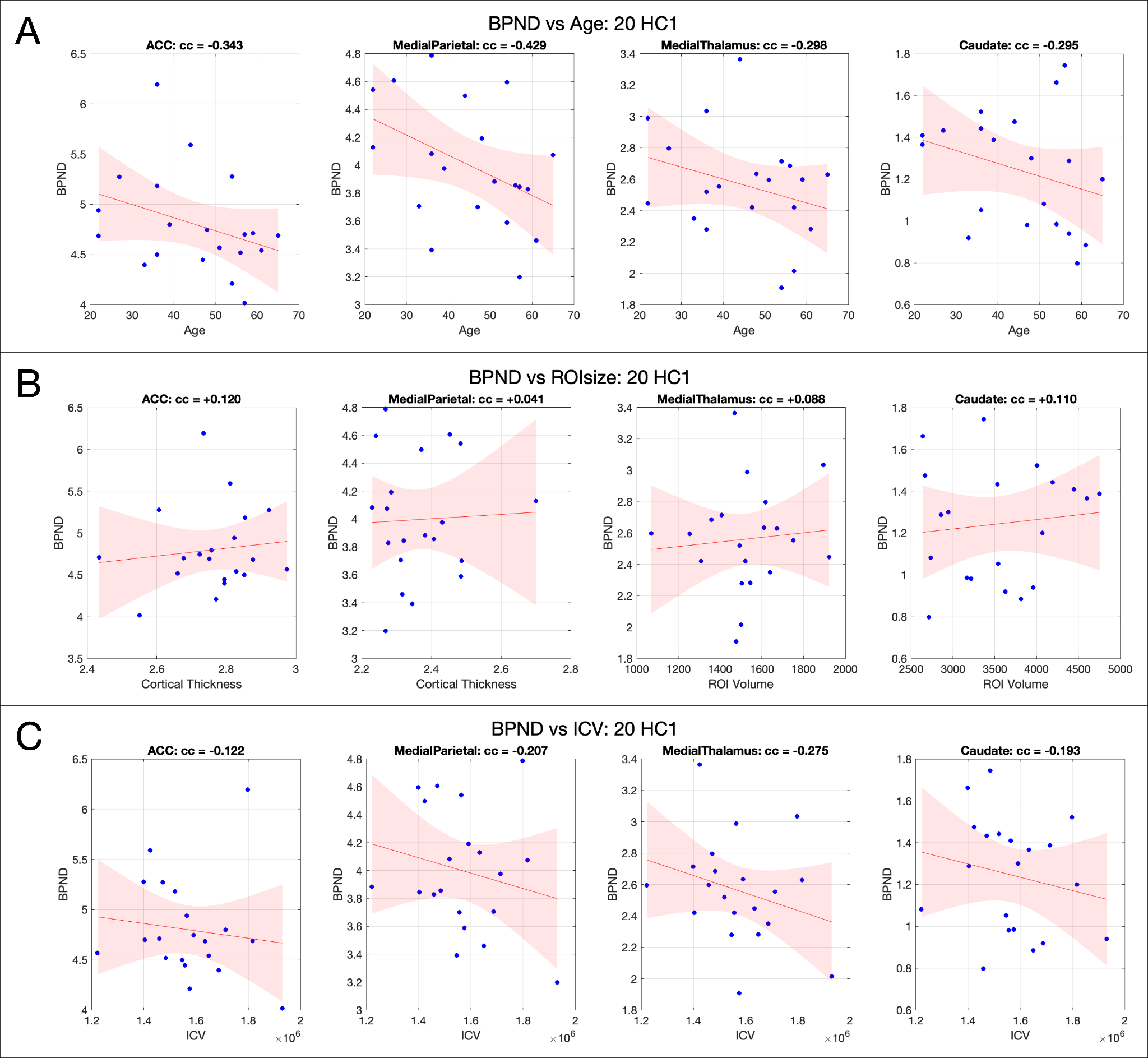


**Supplementary Figure 2: Relationship of healthy control (HC) binding potential (**BP_ND_**) with age, ROI size and total intracranial volume (ICV):** Scatter plots showing the Pearson correlation (cc) between a) Binding potential (BP_ND_) in healthy controls (HC1, baseline scan) and age, b)BP_ND_ and cortical thickness/subcortical volume (ROI size), and c) BP_ND_ and total intracranial volume (ICV). Line-of-best fit (red line) with 95% confidence limits (shaded red region) are shown.


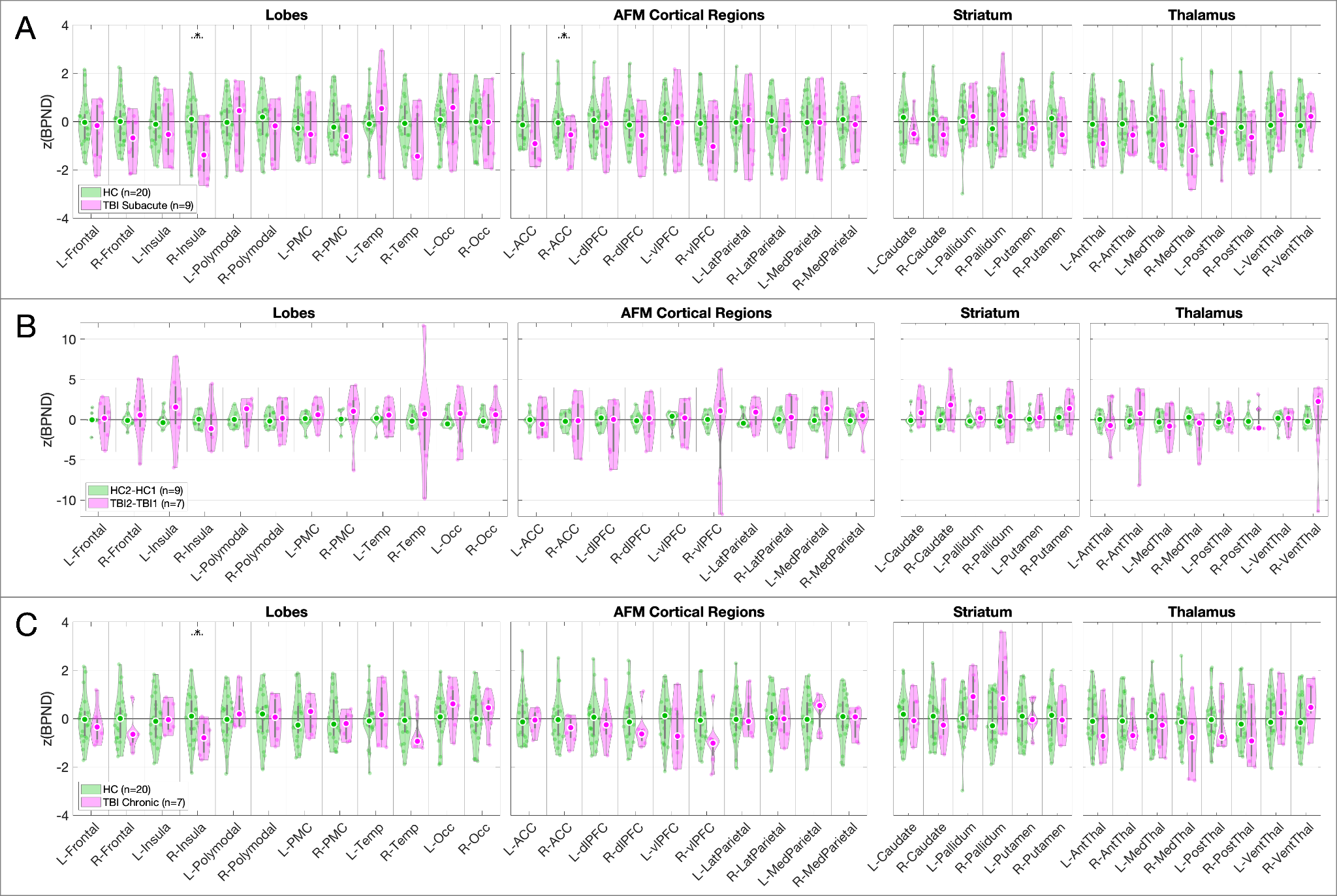


**Supplementary Fig 3. Distribution of regional [^11^C]flumazenil tracer BP_ND_ in individuals with TBI compared to HCs**. **A:** Group differences in [^11^C]flumazenil tracer BP_ND_ in individuals with TBI at sub-acute timepoint (n=9) and control individuals without TBI (n=20). All regional values were z-scored after adjusting for age and cortical thickness/subcortical volume based on healthy controls.. *Represents group differences with HC as the reference (unpaired t-test, uncorrected p<0.05). Most cortical and subcortical regions demonstrate lower mean BP_ND_  for subjects with TBI compared to controls (significant for insula, right anterior cingulate cortex [ACC]) **B:** Longitudinal changes in [11C]flumazenil tracer BP_ND_ in adults with TBI compared to between-scan changes in control individuals without TBI. Changes in BP_ND_ have been adjusted for cortical thickness/subcortical volume differences between the groups. Groups show comparable mean variability across cortical and subcortical regions with marked increase in variation in subjects with TBI across most regions. **C:** Group differences in [^11^C]flumazenil tracer BP_ND_ in individuals with TBI at chronic timepoint (n=7) and control individuals without TBI (n=20). Relative normalization of TBI and control BP_ND_ is evident across most cortical and subcortical structures. Persistently lower BP_ND_ is demonstrated in right insula for TBI; subjects with TBI show marked increases of pallidal BP_ND_. Also see Supplementary Figure 3 for both sub-acute and chronic distributions together.


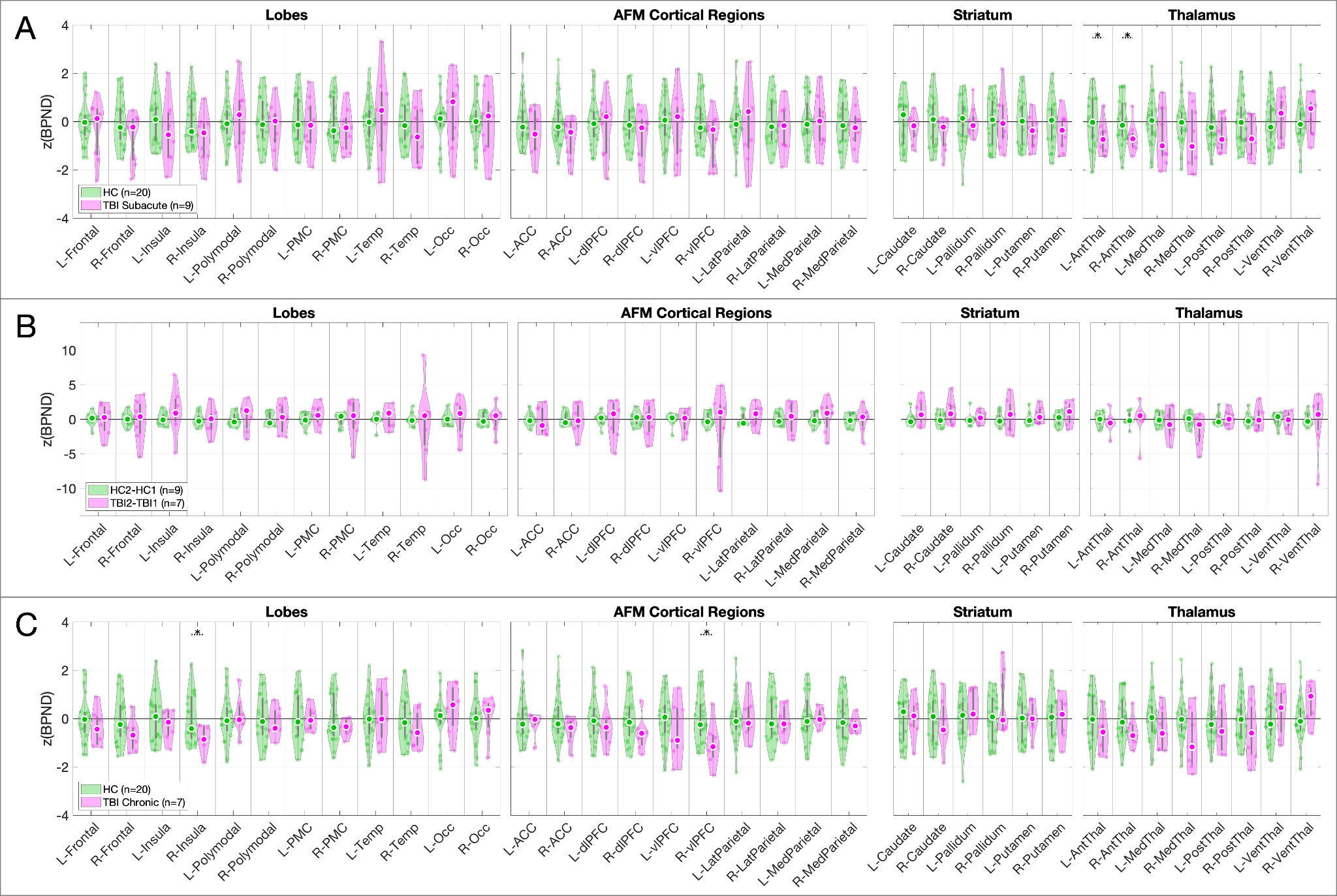


**Supplementary Figure 4: Distribution of regional [^11^C]flumazenil tracer BP_ND_ in individuals with TBI compared to HCs**. **A:** Group differences in [^11^C]flumazenil tracer BP_ND_ in individuals with TBI at sub-acute timepoint (n=9) and control individuals without TBI (n=20). All regional values were z-scored based on healthy controls, but not adjusted for age and cortical thickness/subcortical volume. *Represents group differences with HC as the reference (unpaired t-test, uncorrected p<0.05). Most cortical and subcortical regions demonstrate lower mean BP_ND_  for subjects with TBI compared to controls (significant for left and right anterior thalamus) **B:** Longitudinal changes in [11C]flumazenil tracer BP_ND_ in adults with TBI compared to between-scan changes in control individuals without TBI. Changes in BP_ND_ have been z-scored bvased on healthy controls but not adjusted for age or cortical thickness/subcortical volume. Groups show comparable mean variability across cortical and subcortical regions with marked increase in variation in subjects with TBI across most regions. **C:** Group differences in [^11^C]flumazenil tracer BP_ND_ in individuals with TBI at chronic timepoint (n=7) and control individuals without TBI (n=20). Relative normalization of TBI and control BP_ND_ is evident across most cortical and subcortical structures. Persistently lower BP_ND_ is demonstrated in right insula and right vlPFC for TBI;

**
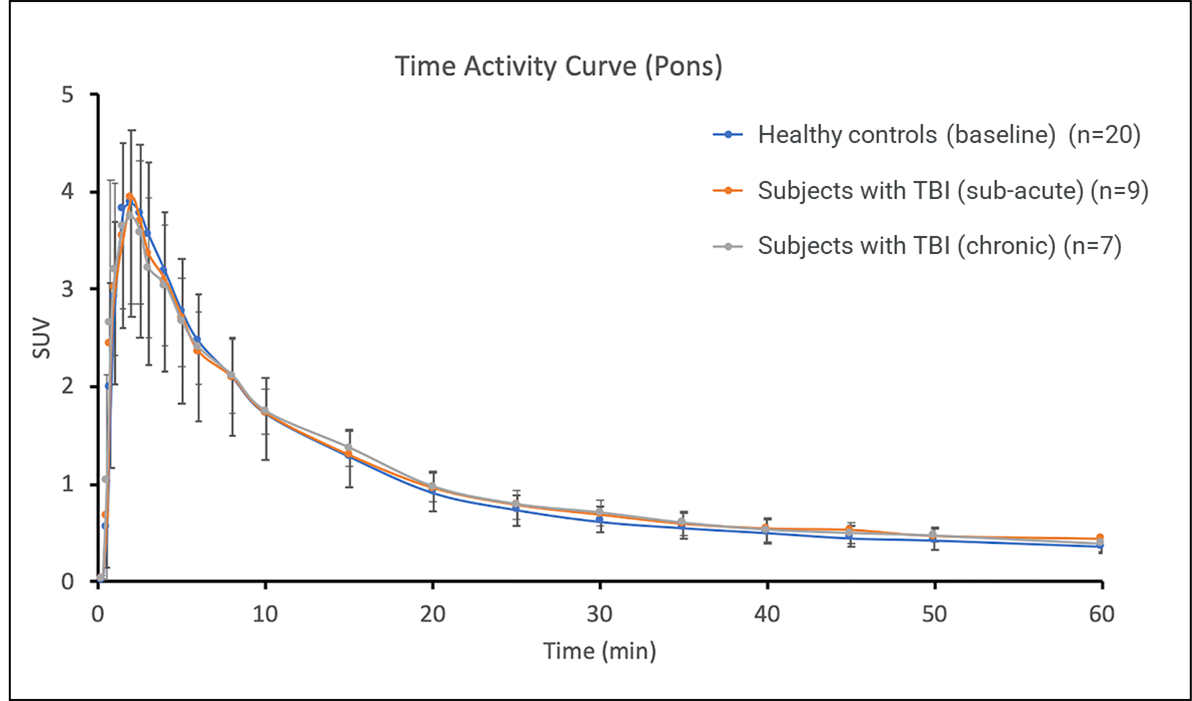
**

**Supplementary Figure 5: Standardized uptake value (SUV) time activity curves in the Pons for healthy controls and subjects with TBI.**


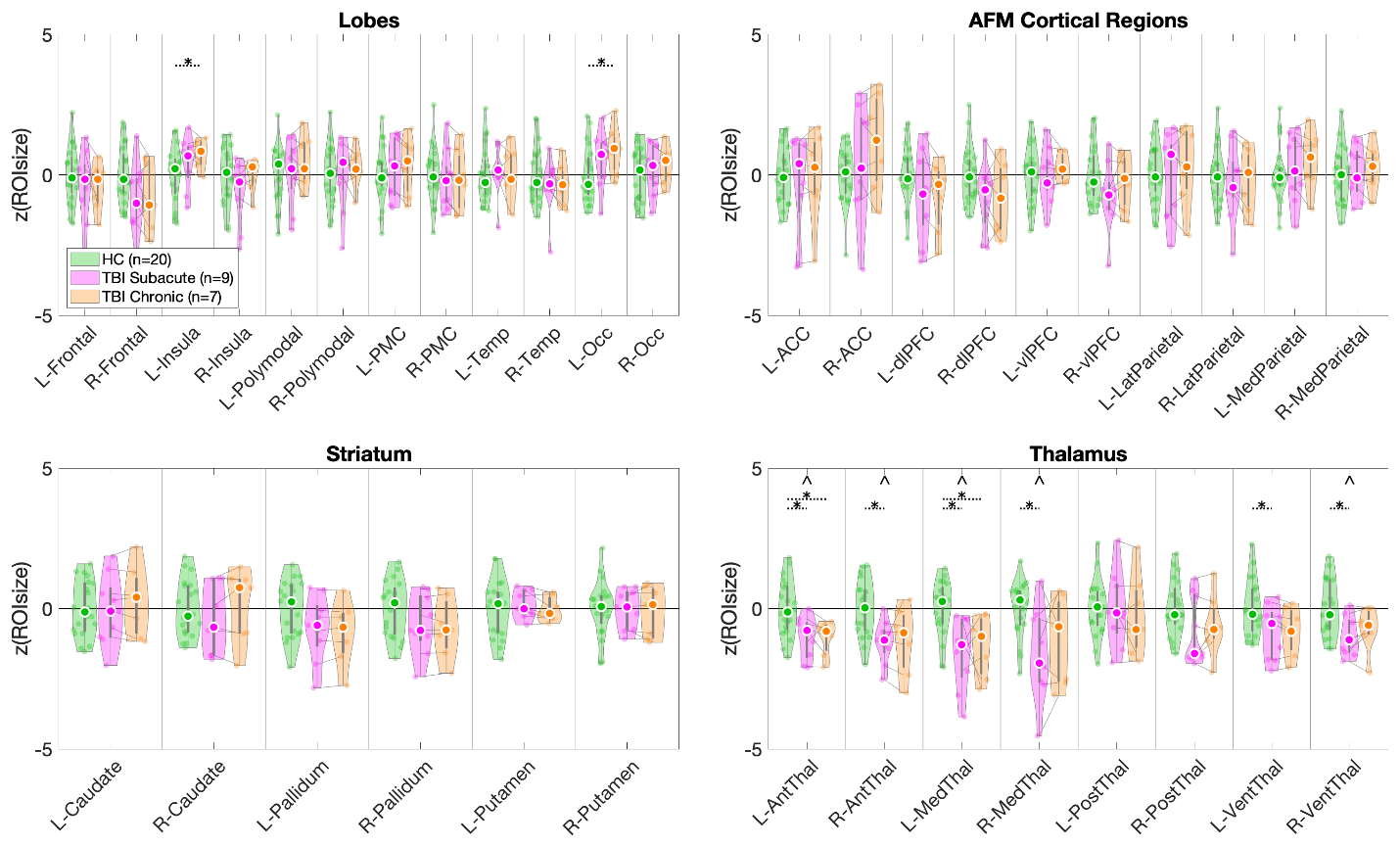


**Supplementary Figure 6.** **Distribution of cortical thickness-subcortical volume in individuals with TBI compared to baseline scan in HCs**. **A:** Group differences in cortical thickness-subcortical volume in individuals with TBI at sub-acute timepoint (n=9), chronic timepoint (n=7) and control individuals without TBI (n=20). Values are z-scored and adjusted for age and total intracranial volume based on healthy controls. *Represents group differences with HC as the reference (unpaired t-test, uncorrected p<0.05). ^Represents group differences (one-way ANOVA, uncorrected p<0.05).

**
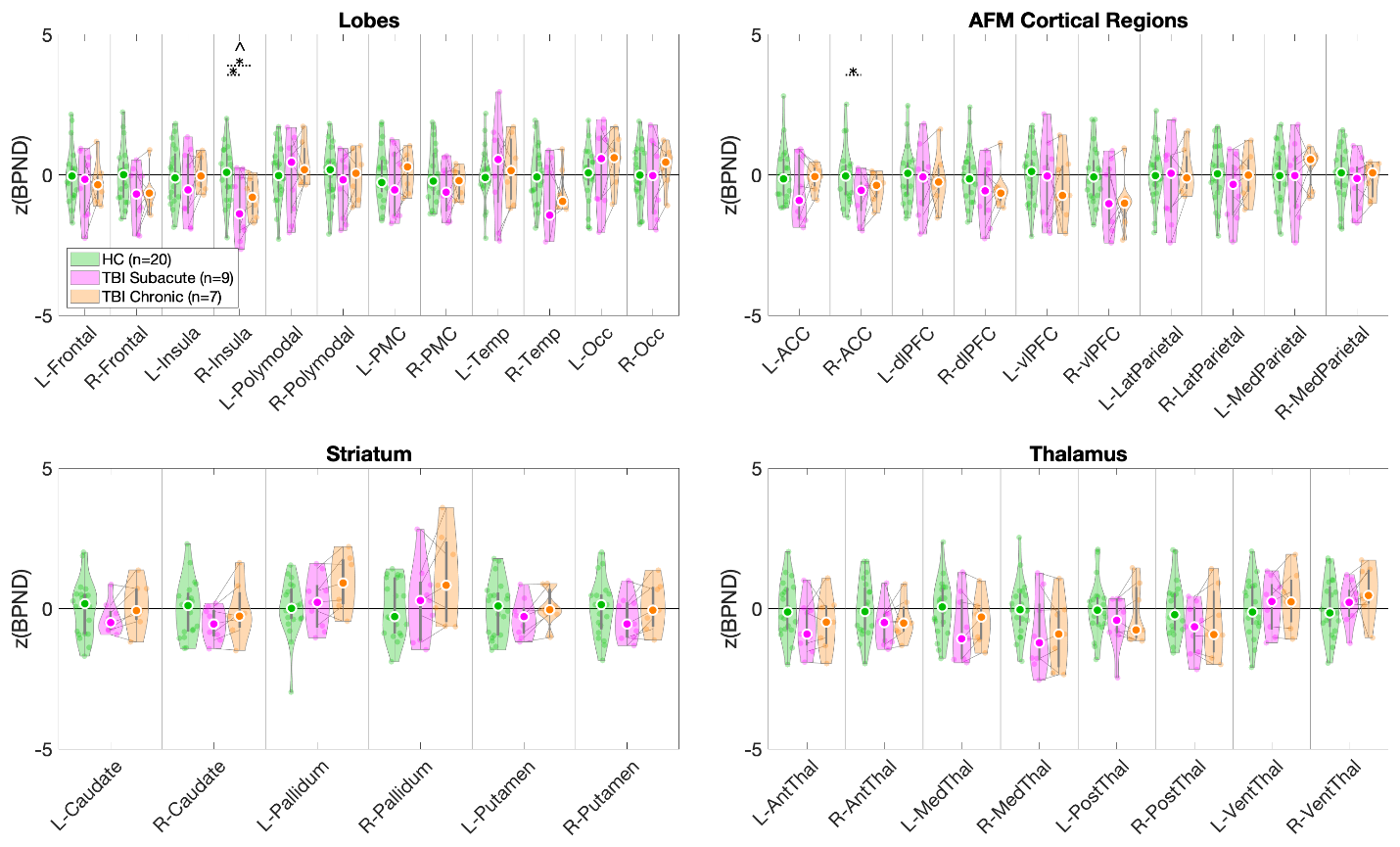
**

**Supplementary Figure 7.** **Distribution of BP**_ND_ **in individuals with TBI compared to baseline scan in HCs**. **A:** Group differences in BP_ND_ in individuals with TBI at sub-acute timepoint (n=9), chronic timepoint (n=7) and control individuals without TBI (n=20). All regional values were z-scored after adjusting for age and cortical thickness/subcortical volume based on healthy controls. *Represents group differences with HC as the reference (unpaired t-test, uncorrected p<0.05). ^Represents group differences (one-way ANOVA, uncorrected p<0.05).

#
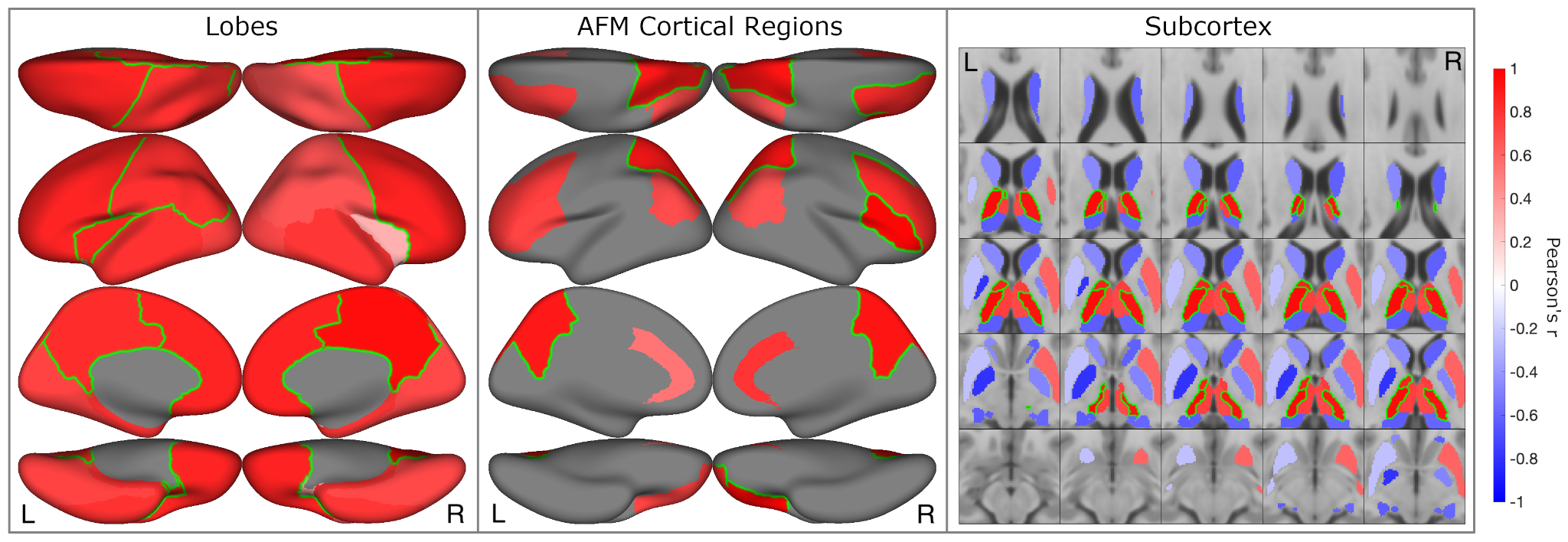


**Supplementary Figure 8: Correlation of change in BP_ND_ in subjects with TBI (n=6) with change in ANT executive behavior z-score.** Significant correlations between global increases in BP_ND_ and behavioral improvements in executive attention (measured by ANT performance) are seen across most cortical regions and bilaterally across the anterolateral thalami. Results have been adjusted for age and cortical thickness/subcortical volume. Excluded 1 subject from both sub-acute and chronic because behavioral result was outlier.

**Supplementary tables**

| ID | Age | Sex | Acute Hospital Length of Stay (days) | Visit 1: Months Since Injury | Visit 2: Months Since Injury | Cause of Injury | Glasgow Coma Scale - Initial | Visit 1: Glasgow Outcome Scale - Extended | Visit 2: Glasgow Outcome Scale - Extended |
| --- | --- | --- | --- | --- | --- | --- | --- | --- | --- |
| 1 | 18 | M | 14 | 4.1 |  | Fall | 5 | 4 |  |
| 2 | 26 | M | 35 | 4.0 |  | Motor vehicle accident | 3 | 4 |  |
| 3 | 58 | M | 5 | 4.8 | 13.0 | Motorcycle accident | 14 | 5 | 7 |
| 4 | 58 | M | 2 | 4.0 | 12.8 | Fall | 14 | 6 | 7 |
| 5 | 48 | M | 5 | 3.4 | 14.6 | Assault with blunt instrument | 14 | 4 | 4 |
| 6 | 33 | F | 9 | 7.0 | 20.4 | Fall | 8 | 7 | 8 |
| 7 | 38 | M | 1 | 4.4 | 15.3 | Motorcycle accident | 12 | 7 | 8 |
| 8 | 49 | F | 7 | 4.5 | 14.4 | Pedestrian accident | 15 | 6 | 7 |
| 9 | 55 | M | 24 | 4.1 | 10.9 | Bicycle accident | 3 | 8 | 8 |

**Supplementary Table 1. Participant demographic data for the subjects with TBI**

|  | **subjects with TBI** | | **Healthy control subjects** | |
| --- | --- | --- | --- | --- |
|  | **Visit 1 (n=9)** | **Visit 2 (n=7)** | **Visit 1 (n=20)** | **Visit 2 (n=9)** |
| **Number (male/female)** | 7/2 |  | 12/8 |  |
| **Age (years)** | 48.0 (18-58) |  | 47.5 (22-65) |  |
| **Time since injury (months)** | 4.1 (3-6) | 14.4 (11-20) | N/A |  |
| **Time between scans (months)** |  | 9.9 (7-13) |  | 5.7 (5-11) |
| **Glasgow Coma Scale - Initial** | 11 (3-15) |  | N/A |  |
| **Glasgow Outcome Scale-Extended** | 6 (4-8) | 7 (4-8) |  |  |
| **Acute hospital length of stay (days)** | 8 (2-35) |  | N/A |  |
| **FMZ Dosage (MBq)** | 535 (420-611) | 539 (487-601) | 546 (487-625) | 502 (427-563) |

**Supplementary Table 2. Participant demographic data for the TBI and healthy control cohorts.** Values are shown as median and (range).

| **LOBES** | |
| --- | --- |
| Frontal Lobe | precentral, superior frontal, caudal middle frontal, rostral middle frontal, pars opercularis, pars triangularis, lateral orbitofrontal, medial orbitofrontal, caudal anterior cingulate, rostral anterior cingulate, frontal pole |
| Insular Cortex | insula |
| Polymodal Cortex | postcentral, superior parietal, inferior parietal, supramarginal |
| Posteromedial Cortex (PMC) | precuneus, isthmus cingulate, posterior cingulate, paracentral |
| Temporal Lobe | superior temporal, middle temporal, inferior temporal, transverse temporal, banks of the superior temporal sulcus, temporal pole, entorhinal, parahippocampal |
| Occipital Lobe | cuneus, pericalcarine, lingual, fusiform, lateral occipital |
| **ANTERIOR FOREBRAIN MESOCIRCUIT CORTICAL GROUPS** | |
| Anterior Cingulate Cortex (ACC) | caudal anterior cingulate, rostral anterior cingulate |
| Dorsolateral Prefrontal cortex (dlPFC) | caudal middle frontal, rostral middle frontal |
| Ventrolateral Prefrontal Cortex (vlPFC) | pars opercularis, pars triangularis, pars orbitalis |
| Lateral Parietal | inferior parietal |
| Medial Parietal | precuneus, superior parietal |
| **SUBCORTEX GROUPS** | |
| Anterior Thalamus | Anteroventral (AV), Lateraldorsal (LD), Lateral posterior (LP), Ventral anterior (VA+VAmc) |
| Ventral Thalamus | Ventral lateral (VLa+VLp), Ventral posterolateral (VPL) |
| Posterior Thalamus | Lateral geniculate (LGN), Median geniculate (MGN), Limitans/suprageniculate (L-Sg), Pulvinar (PuA+PuM+PuL+PuI) |
| Medial Thalamus | Mediodorsal (MDm+MDl), Central median (CeM), Central lateral (CL), Paracentral (Pc), Centromedian (CM), Parafascicular (Pf) |
| Caudate | n/a |
| Pallidum | n/a |
| Putamen | n/a |

**Supplementary Table 3. Region of interest definitions.** Cortical groups were derived from the Desikan-Killiany gyral atlas in FreeSurfer.[^4^](https://www.zotero.org/google-docs/?ZMZUzJ) Basal ganglia were derived from the aseg[^5^](https://www.zotero.org/google-docs/?BAekWr) FreeSurfer output, as modified by FSL’s FIRST.[^6^](https://www.zotero.org/google-docs/?KfzEpV) Thalamic nuclei were derived from the FreeSurfer 7.0 probabilistic thalamic nuclei atlas.[^2^](https://www.zotero.org/google-docs/?6DjRrK)


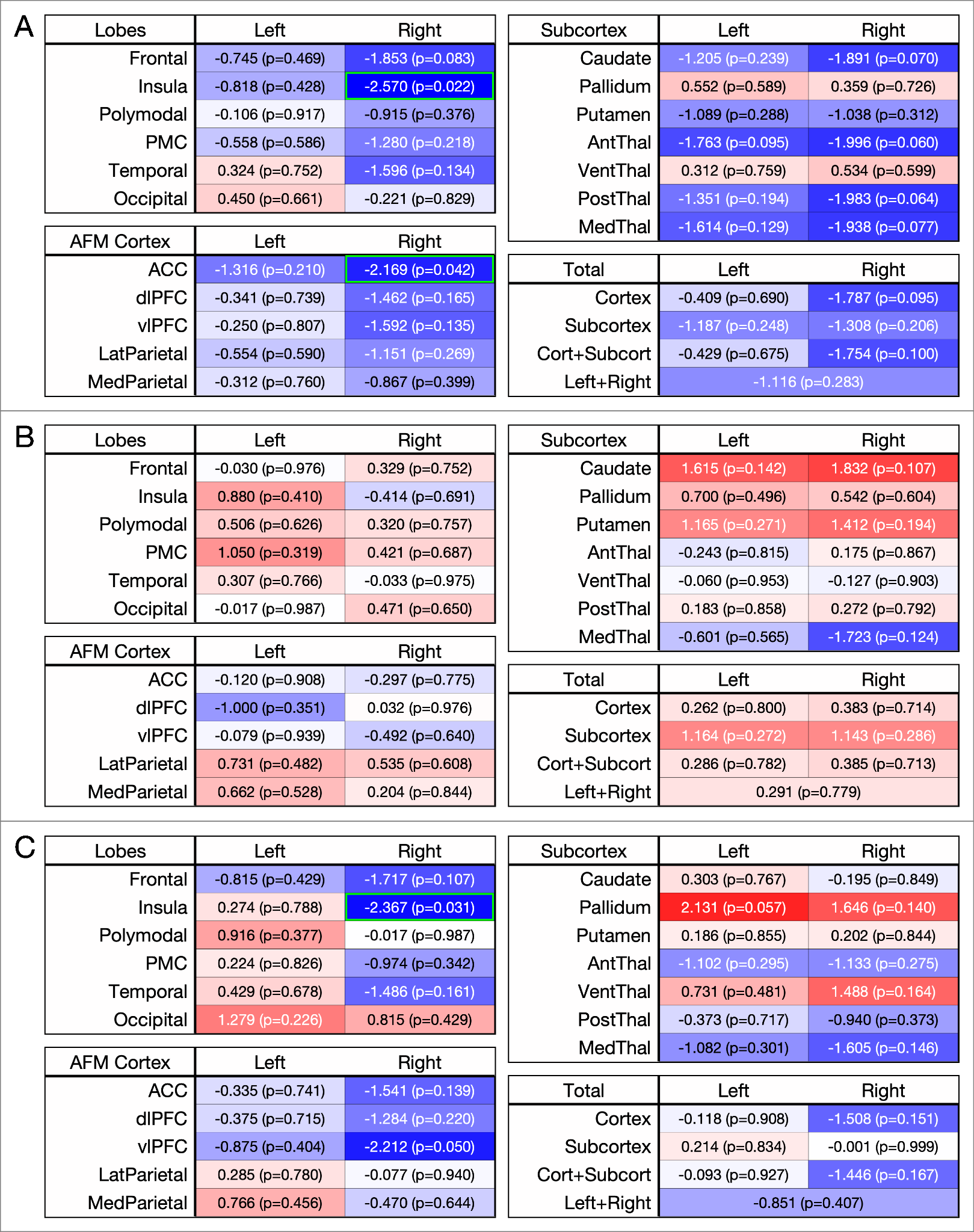


**Supplementary Table 4:** **T-tests for group level differences in [^11^C]flumazenil tracer BP_ND_ between subjects with TBI compared to HC subjects. A:** Controls, n=20, TBI Subacute, n = 9. p-values are uncorrected. **B:** T-values for group differences in [^11^C]flumazenil tracer BP_ND_ change between scans (HC = 9, TBI = 7). **C:** Controls, n=20, TBI chronic, n = 7.

**References**

[1. Kang Y, Rúa SMH, Kaunzner UW, et al. A Multi-Ligand Imaging Study Exploring GABAergic Receptor Expression and Inflammation in Multiple Sclerosis. *Mol Imaging Biol*. 2020;22(6):1600-1608. doi:10.1007/s11307-020-01501-z](https://www.zotero.org/google-docs/?3YB1Id)

[2. Iglesias JE, Insausti R, Lerma-Usabiaga G, et al. A probabilistic atlas of the human thalamic nuclei combining ex vivo MRI and histology. *NeuroImage*. 2018;183:314-326. doi:10.1016/j.neuroimage.2018.08.012](https://www.zotero.org/google-docs/?3YB1Id)

[3. Fan J, McCandliss BD, Sommer T, Raz A, Posner MI. Testing the efficiency and independence of attentional networks. *J Cogn Neurosci*. 2002;14(3):340-347. doi:10.1162/089892902317361886](https://www.zotero.org/google-docs/?3YB1Id)

[4. Desikan RS, Ségonne F, Fischl B, et al. An automated labeling system for subdividing the human cerebral cortex on MRI scans into gyral based regions of interest. *NeuroImage*. 2006;31(3):968-980. doi:10.1016/j.neuroimage.2006.01.021](https://www.zotero.org/google-docs/?3YB1Id)

[5. Fischl B, Salat DH, Busa E, et al. Whole Brain Segmentation. *Neuron*. 2002;33(3):341-355. doi:10.1016/S0896-6273(02)00569-X](https://www.zotero.org/google-docs/?3YB1Id)

[6. Patenaude B, Smith SM, Kennedy DN, Jenkinson M. A Bayesian model of shape and appearance for subcortical brain segmentation. *NeuroImage*. 2011;56(3):907-922. doi:10.1016/j.neuroimage.2011.02.046](https://www.zotero.org/google-docs/?3YB1Id)
